# Genetic polymorphisms in the osteopontin promoter increases the risk of distance metastasis and death in Chinese patients with gastric cancer

**DOI:** 10.1186/1471-2407-12-477

**Published:** 2012-10-16

**Authors:** Fujun Zhao, Xiaoyi Chen, Tingting Meng, Bo Hao, Zhihong Zhang, Guoxin Zhang

**Affiliations:** 1 Department of Gastroenterology, First Affiliated Hospital of Nanjing Medical University, Nanjing, 210029, China; 2 Department of Pathology, First Affiliated Hospital of Nanjing Medical University, Nanjing, 210029, China; 3 Jiangyan People’s Hosptial, Jiangyan City, 225500, Jiangsu Province, China

**Keywords:** Osteopontin, Gastric cancer, Polymorphisms, Genetic variants, Metastasis

## Abstract

**Background:**

*In vitro* and *in vivo* studies have suggested that osteopontin (OPN) is associated with many types of cancers. However, no studies have reported the incidence of OPN polymorphisms and the risk of gastric cancer. The aim of this study was to investigate the association between OPN polymorphisms and gastric cancer in a Chinese patient population.

**Methods:**

Three genetic variants in the OPN promoter were genotyped using direct sequencing in 200 gastric cancer patients and 200 gender- and age-matched cancer-free controls. The 4-year survival curve was calculated using the Kaplan-Meier method and compared using the log-rank test for each single nucleotide polymorphism (SNP) site. We measured the promoter activity of the -443 T → C polymorphism using a dual luciferase reporter assay.

**Result:**

For the variant at nt -443 (CC), there was a significant difference between the number of patients with stage IV and those with stage I gastric cancer (IA + IB; *P =* 0.014) and between those with stage IV and all other stages of gastric cancer (IA + IB + II + III; *P =* 0.02). For the variant at nt -443 (CT), there was a significant difference between the number of gastric cancer patients with stage IV and those with stage II (*P =* 0.013). The survival rates for patients with the C/C genotype were significantly lower than for patients with the other two genotypes (C/T, T/T). Moreover, significantly higher luciferase activities were observed in the pGL3-C construct compared to the pGL3-T construct.

**Conclusions:**

This study provides the first evidence that variation at nt -443 in the OPN promoter increases the potential for gastric cancer metastasis and subsequent death in the Chinese population.

## Background

Gastric adenocarcinoma remains the second leading cause of cancer-related deaths worldwide, accounting for 738,000 deaths annually [1]. Gastric cancer is the third most common cancer in China. The development of gastric cancer is associated predominantly with *Helicobacter pylori* infection [2], but other risk factors include a diet high in salt, smoking, consumption of pickled foods, and specific genetic backgrounds [3]. It has been shown that *H. pylori* infection is an independent risk factor that leads to persistent colonization and chronic inflammation of the gastric mucosa, thereby increasing the risk of developing peptic ulceration and gastric cancer [2,4-6]. However, there are marked inter-individual differences in the extent of inflammation among persons with *H. pylori* infection, and clinical consequences only develop in a small numbers of gastric cancer cases. Multifactorial models suggest that the genetic susceptibility due to specific variant alleles in polymorphisms may affect the outcomes of environmental exposure [7].

Osteopontin (OPN) is a secreted adhesive phosphoglycoprotein that contains a functional Gly-Arg-Gly-Asp-Ser cell-binding sequence [8]. The *OPN* gene has been mapped to chromosome 4q24-q25, and it has been shown that OPN plays an important role in tumor metastasis [9]. OPN has been shown to be expressed within tumor cells and in the surrounding stroma of numerous human cancers, such as colon, breast, lung, stomach, endometrium, and thyroid, providing a link with malignant invasion [10-13]. Previous studies showed that OPN is frequently overexpressed in human gastric cancer [14], and that expression of OPN mRNA was significantly higher in gastric cancer tissues compared to non-tumor tissues.

Several polymorphisms have been described for the *OPN* gene, some of which are associated with oligoarticular (or pauciarticular) juvenile idiopathic arthritis, nephrolithiasis, and chronic hepatitis C [15-18]. A recent study [19] reported that patients with a G/G genotype at nt -156 in the OPN promoter were more frequently diagnosed with advanced stage (IIIB-IV) non-small cell lung cancer (NSCLC) than those with other genotypes, while another report suggested that the OPN polymorphism might be the genetic factor for hepatitis B viral clearance and hepatocellular carcinoma occurrence [20].

There are currently no published studies assessing the relationship between *OPN* genetic polymorphisms and the risk of gastric cancer development. Therefore, the aim of this study was to determine if an association exists between OPN polymorphisms and the risk of gastric cancer in the Chinese population.

## Methods

**Subjects** From 2005 to 2008, 310 unrelated patients with gastric cancer (the GC group), were enrolled at the First Affiliated Hospital of Nanjing Medical University. All of the patients were ethnic Han Chinese residents who had histologically confirmed gastric adenocarcinoma. The control group (the non-GC group) consisted of a random sample of 591 ethnic Han Chinese from Jiangsu Province. After giving written informed consent, all participants were requested to provide a blood sample. This study was approved by the Ethics Committee of the First Affiliated of Nanjing Medical University (# 2010-SR-073).

Before sequencing the genotypes in the *OPN* promoter, we used SPSS v10.0 (SPSS, Inc., Chicago, IL, USA) software to randomly select 200 GC patients and gender- and age-match them to 200 randomly selected controls of the non-GC group. We evaluated all patients and controls for *H. pylori* using an indirect solid phase immunochromatographic (ICM) assay to investigate the presence of IgG antibodies to *H. pylori* (Genelabs Diagnostics, Singapore). This test method was previously validated in our lab with an accuracy of 92.3% [21].

Genomic DNA from controls and gastric cancer patients was extracted from ethylenediaminetetracetic acid (EDTA)-anticoagulated peripheral blood according to the traditional proteinase K and phenol-chloroform method, and stored at -70°C.

### Analysis of polymorphisms in the OPN regulatory region

The OPN-66, -156(rs17524488), and -443(rs11730582) variants were genotyped by direct sequencing of the sense and anti-sense strands following polymerase chain reaction (PCR) amplification of the promoter regulatory region -473 to -3 (forward primer 5^′^-CAA GCT ACT GCA TAC TCG AAA TCA CA-3^′^; reverse primer 5^′^-ACA ACC AAG CCC TCC CAG AAT TTA-3^′^), as previously described [19]. PCR was performed using 50 ng DNA as a template under the following conditions: 95°C for 10 min, then 36 cycles of 94°C for 30 s, an annealing temperature for 60 s, and 72°C for 60 s, with a final extension at 72°C for 15 min. After affinity membrane purification using the QIAquick Gel Extraction kit (Qiagen, Carlsbad, CA, USA), the PCR products were subjected to cycle sequencing with the respective forward and reverse primer using an automated ABI 3100 DNA sequencer by GeneCore Bio Technologies (Shanghai China).

### Luciferase assay with SNP at nucleotide (nt) -443 in the OPN promoter

The 250 bp fragments of the OPN promoter (from -590 to -340) carrying either the T or C allele were synthesized by Invitrogen and inserted upstream of the firefly luciferase gene in the pGL3-Enhancer plasmid vector (Promega, USA) in separate procedures. Each construct was confirmed by sequencing. A series of OPN promoter-luciferase reporter constructs were transiently transfected into the gastric cancer cell lines MKN28 (Shanghai Institute of Digestive Disease) and SGC-7901 (Shanghai Cancer Institute), and transfection was performed with Lipofectamine 2000 Reagent (Invitrogen) according to the manufacturer’s protocol. The MKN28 and SGC-7901 cell lines were cultured in Dulbecco’s modified Eagle’s medium (DMEM) supplemented with 10% heat-inactivated fetal bovine serum. The activity of the different genotype promoters was measured using the Luciferase Reporter Assay System (Promega, USA). All experiments were repeated in triplicate.

### Statistical analysis

Statistical analysis was performed using SPSS 10.0 software. Quantitative variables departing from the normal distribution, including age, were summarized as mean and standard deviation (SD). Comparison of age between cases and controls was assessed using an independent Student’s *t*-test. Comparison of extra-gastric tumors, *H. pylori* infection, and genotype frequencies between cases and controls was assessed using a chi-square test and a Fisher’s exact test. Survival was calculated by the Kaplan-Meier method. All probability (*P*) values were two-tailed and statistical significance was indicated as *P* < 0.05.

## Results

### Demographic and clinical features of the patients

The gastric cancer (GC) group consisted of 200 individuals (64% male), with a mean age of 56.29 ± 3.46 years. The control (non-GC) group consisted of 200 individuals, of which 64% were male, with a mean age of 55.67 ± 4.21 years (Table 1). There were no significant differences in terms of distribution of age and gender as well as *H. pylori* seropositivity. Clinicopathologic characteristics of the patients and controls are shown in Table 1.

**Table 1:** Clinicopathologic characteristics of patients with gastric cancer carcinoma and healthy controls

**Characteristic**	**No. of patients or controls**		***P***
	**Cases (n)**	**Control (n)**	
No.	200	200	
Age, years			> 0.05
Mean	56.29	55.67	
Standard deviation	3.46	4.21	
Range	63	65	
Gender			> 0.05
Male	130	130	
Female	70	70	
*Helicobacter pylori* infection			0.12
Seronegative	62	77	
Seropositive	138	123	
Vascular invasion			
Absence	155	-	
Presence	45	-	
Lymph node metastasis			
Absence	80	-	
Presence	120	-	
Liver metastasis			
Absence	182	-	
Presence	18	-	
Peritoneal dissemination			
Absence	172	-	
Presence	28	-	
TNM stage		-	
IA	39	-	
IB	40	-	
II	33	-	
III	45	-	
IV	43	-	

### SNPs in the promoter region of human OPN gene

**Figure 1:**
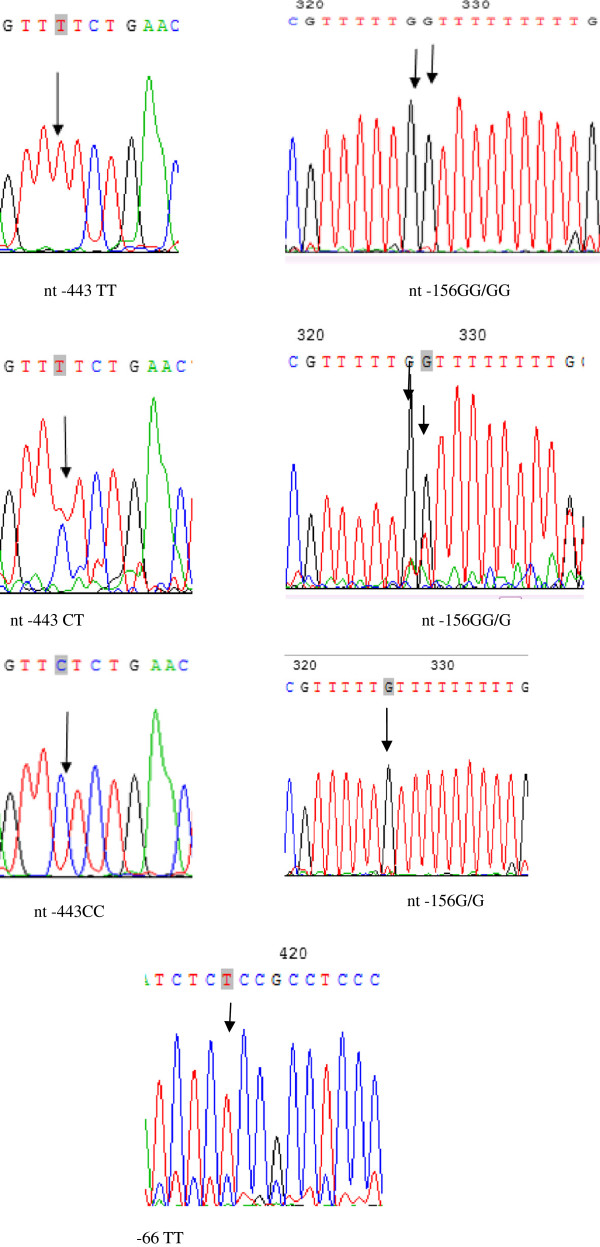
**Schematic diagram and sequencing data of the OPN promoter.** Representative figure for the sequencing analysis on the promoter. The SNP nt -443 has the following alleles: CC, CT, and TT. There is a small insertion at nt-156, which has three alleles: G/G, G/GG, GG/GG. The SNP nt -66 has only one allele: TT.

Direct sequencing of DNA fragments between nt -473 and nt -3 in patients and age- and gender-matched controls revealed 3 SNPs in the OPN promoter, located at nt -156 [GG/GG homozygotes, GG/G-(deletion) heterozygotes, G-/G- homozygotes], nt -443 [CC homozygotes, CT heterozygotes, TT homozygotes], and nt -66 (Figure 1), as shown in Table 2. There was no significant difference in the distribution of these SNPs (nt -66, -156, -443) between GC patients and controls. The distribution of genotypes for TNM stages in gastric cancer is shown in Table 3.

**Table 2:** Comparison of OPN promoter between gastric cancer patients and healthy controls

	**Controls**	**Patients**		**Gastric cancer**		
**Genotypes**	**n**	**n**	***P***	**LN (+)**	**LN (-)**	***P***
-66						
TT	200	200	1.00	124	76	1.00
-156						
G/G	86	67	1.00	41	25	1.00
G/GG	78	92	0.064	57	36	0.92
GG/GG	36	41	0.18	25	16	0.91
-443						
CC	22	15	1.00	8	8	1.00
CT	93	94	0.28	63	33	0.23
TT	85	91	0.22	53	35	0.45

**Table 3:** The distribution of genotypes for TNM stages in gastric cancer

	**The TNMs of gastric cancer**				
**Genotypes**	**IA**	**IB**	**II**	**III**	**IV**
-66					
TT	38	44	26	52	40
-156					
G/G	16	16	13	20	19
G/GG	16	15	15	16	18
GG/GG	7	9	5	9	6
-443					
CC	1	2	1	4	14
CT	17	19	19	19	19
TT	14	18	5	19	29

However, regarding tumor-node-metastasis TNM stages, we found that for the SNP at nt -443, among patients with the CT genotype, there was a significant difference between patients with stages II and IV (*P* < 0.01), and between stage IV and all other stages (IA + IB + II + III; *P =* 0.04; Table 4). Similarly, among patients with the CC genotype at nt -443, there was a significant difference between patients with stages IV and stage I (IA + IB; *P =* 0.011) and between stage IV and all other stages (IA + IB + II + III; *P* = 0.012; Table 4). There were no significant differences among the TNM stages and the other two SNPs (nt -66 and nt -156) of the OPN promoter. We also found no association between the SNPs in the OPN promoter and lymph node metastasis.

**Table 4:** The genotype distribution of nt -443 in the OPN promoter by gastric cancer TNM stage

	**The TNM stages of gastric cancer**											
**Genotypes**	**IA + IB**	**IV**	***P***	**II**	**IV**	***P***	**III**	**IV**	***P***	**IA + IB + II + III**	**IV**	***P***
-443												
TT	32	29	1.00	5	29	1.00	19	29	1.00	56	29	1.00
CT	36	19	0.16	19	19	< 0.01*	19	19	0.33	74	19	0.04*
CC	3	14	0.011*	1	14	0.98	4	14	0.18	8	14	0.012*

* indicates significant difference (P < 0.05).

### Associations between genotypes in the OPN promoter region and survival

**Figure 2:**
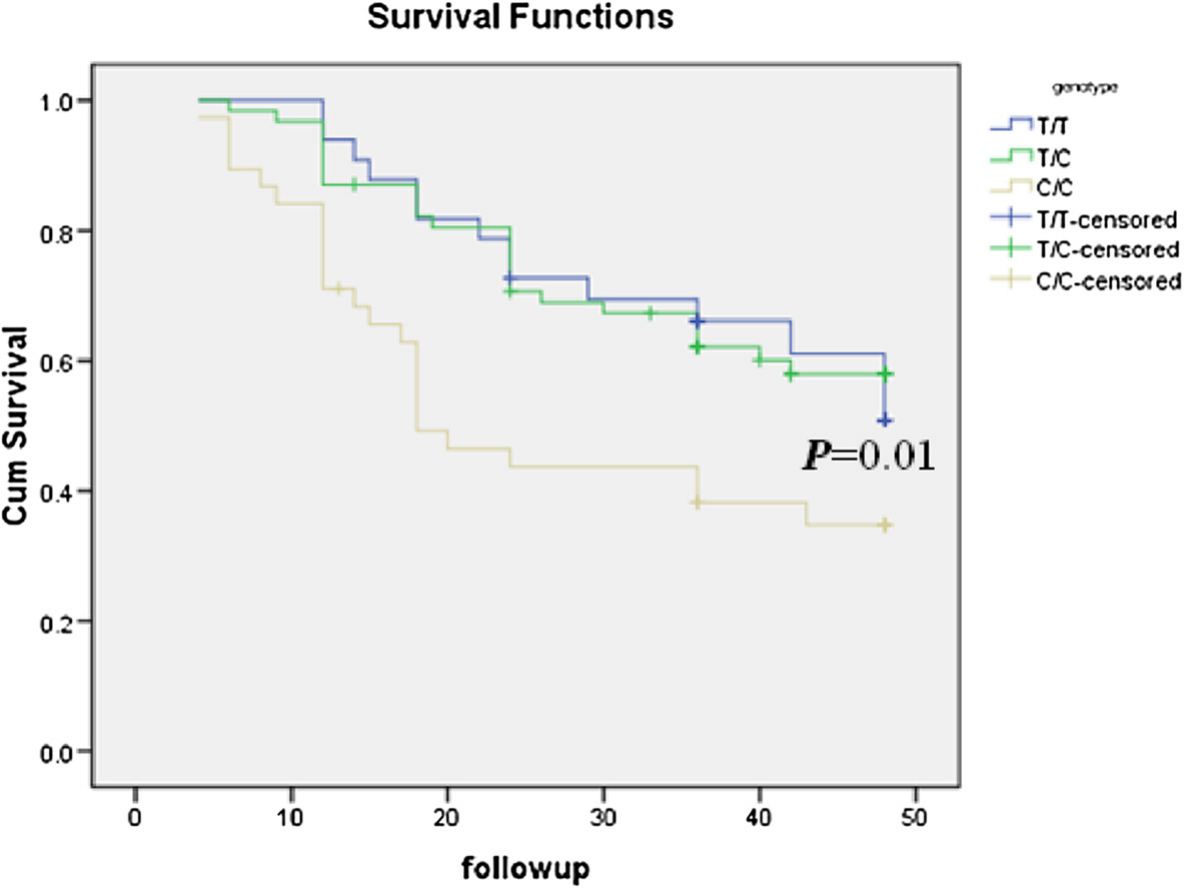
Kaplan-Meier survival is significantly lower in gastric cancer patients with the C/C genotype as compared to the other two genotypes at nt -443 in OPN promoter.

Kaplan-Meier estimates of different genotypes at nt -443 in the *OPN* promoter are shown in Figure 2. The survival rates for patients with the C/C genotype were significantly lower than the survival rates for patients with the other two genotypes (C/T, T/T). There were no significant associations between survival and genotypes at the other sites (nt -156 and nt -66).

### Effect of the -443 T → C polymorphism on promoter activity

**Figure 3:**
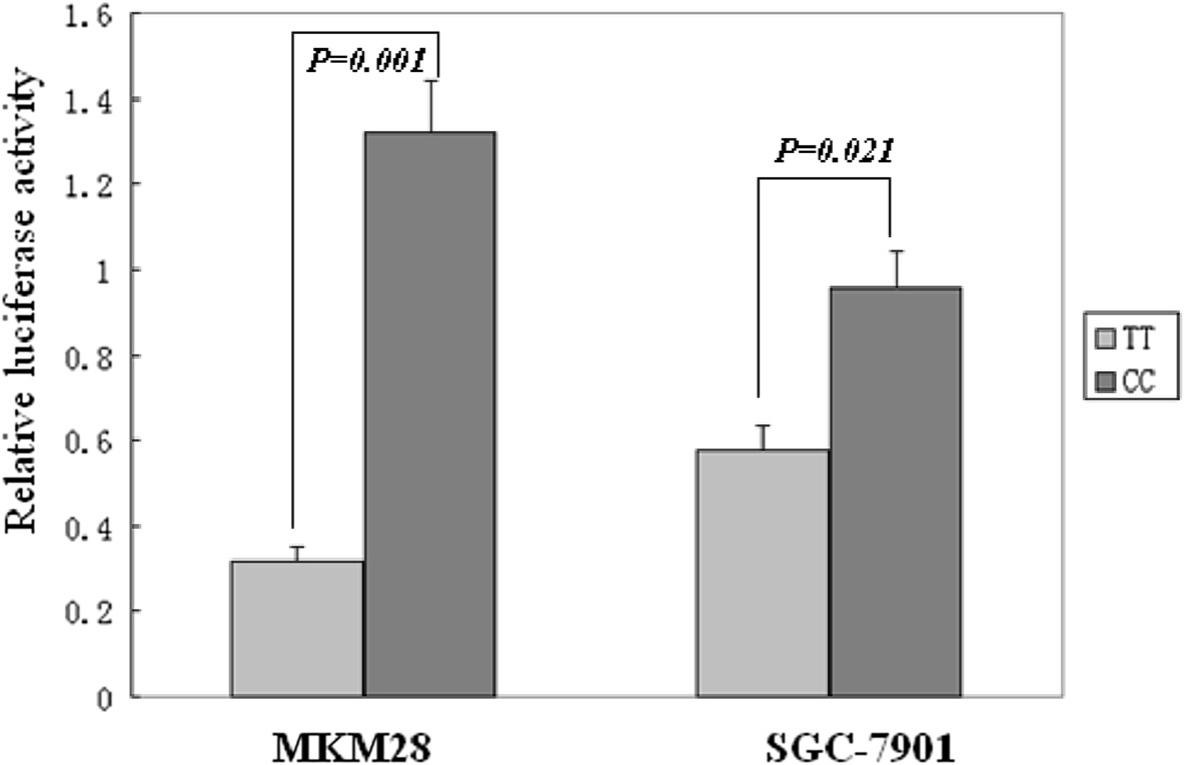
**Effect of the -443 T → C polymorphism on promoter activity.** Significantly higher luciferase activities were generated by the pGL3-C construct as compared with the pGL3-T construct (*P* = 0.001 for MKN28; *P* = 0.021 for SGC-7901).

To examine the effect of the -443 T → C promoter region polymorphism on transcription of the *OPN* gene, we measured promoter activity with a Dual Luciferase Reporter Assay System and compared the activities of the -443C and -443 T alleles using a transient transfection assay with the MKN28 and SGC-7901 cell lines. As shown in Figure 3, significantly higher luciferase activities were generated with the pGL3-C construct compared to the pGL3-T construct (*P* = 0.001 for MKN28; *P* = 0.021 for SGC-7901).

## Discussion

Mounting evidence suggests that OPN plays a role in the regulation of tumor metastasis and that OPN expression is particularly high in metastatic tumors [22]. OPN is overexpressed in cancers that have a high propensity for forming bone metastases. In bone metastases, OPN is generally associated with the interface between the carcinoma and the bone surface, and this appears to be related to increased bone resorptive activity by osteoclasts [23]. Moreover, high OPN expression in the primary tumor is associated with early metastasis and poor clinical outcome in human gastric cancer and other cancers [24-27].

A recent study suggested that the OPN promoter was associated with NSCLC [19]. In the present study, we focused on the association of these SNPs with GC, and although the distribution of genotypes in the *OPN* promoter was not significantly different between GC patients and healthy controls, there were significant differences in the distribution of genotypes (CC) at nt -443 between patients with stage IV and stage I gastric cancer (IA + IB) and between stage IV and the combined other three stages of gastric cancer (IA + IB + II + III; Table 4). The survival rates for patients with the C/C genotype were significantly lower than the survival rates of the other two genotypes (C/T, T/T; Figure 3). In addition, significantly higher luciferase activities were generated with the pGL3-C construct compared to the pGL3-T construct. Reporter gene analysis has shown that the haplotype -443C/-156 G/-66 T is associated with significantly enhanced promoter activity compared to five other allelic variants tested [28]. A recent study on melanoma metastases found that those homozygous for the -443C allele expressed significantly higher levels of OPN mRNA compared to those that were either heterozygous (CT) or homozygous for the -443 T allele [29]. Transcription factor c-Myb binds to the region of the OPN promoter in an allele-specific manner and induces enhanced activity of the -443C compared to the -443 T *OPN* promoter [30]. Taken together, these data suggest that the variation at nt -443 in the OPN promoter plays a role in GC progression and metastasis, especially for the CC genotype at nt -443 in the *OPN* promoter. Whether the polymorphisms of OPN is related to expression of OPN in cancer patients remain unknown although. Over-expression of OPN was found in gastric cancer samples in a previous study [14]. Therefore, additional studies are needed to further elucidate this finding.

In the present study, we found that the CT genotype at nt -443 in the OPN promoter showed significant differences between stage IV and stage II gastric cancer, and also between stage IV and other stages of gastric cancer (IA + IB + II + III), but not between stage IV and stage III or stage I. The main reason for this may be due to the limited number of patients in each subgroup. It is also possible that the transcription factor c-Myb might have enhanced the activity of the region of the *OPN* promoter that contained the CC or CT genotypes, but not the other genotype (i.e., TT) [29]. However, these hypotheses require further investigation in larger studies.

The present genomic findings in healthy controls were not identical to previous findings among Japanese and Italian control subjects [30,31]. Although previous reports suggest that high OPN is expressed at high levels in GC [17], we found no association between the genotypes of the *OPN* promoter with the risk of GC. However, we have found ethnic differences in SNPs of several host genes in GC patients [30,31]. Therefore, the present findings may not apply to all populations. Nonetheless, although there was no association between OPN SNPs and GC gastric cancer susceptibility or severity in Chinese patients, our findings do suggest that there is an association with metastasis of GC.

## Conclusion

In conclusion, this is the first study of OPN genetic polymorphisms and the risk of GC in a Chinese population. We have demonstrated that genetic polymorphisms at -443 in the OPN promoter are associated with metastasis and subsequent death of GC. Therefore, these findings may offer an approach to predict the clinical outcome of GC patients. However, additional studies are needed using a larger cohort of patients in order to confirm these findings.

## Competing interests

The authors declare no competing interests.

## Authors’ contributions

ZF participated in the design of the study and performed the statistical analysis, CX carried out the Luciferase assay, MT, HB conceived of the study, and particpated in its design and coordination, ZZ, ZG participated in the design of the study. All authors read and approved the final manuscript.

## Pre-publication history

The pre-publication history for this paper can be accessed here:

http://www.biomedcentral.com/1471-2407/12/477/prepub
